# Bedside open tracheostomy in COVID-19 patients - a safe and swift approach

**DOI:** 10.4317/medoral.26326

**Published:** 2023-11-22

**Authors:** Nur Wahidah Wahid, Peter Deutsch, Aakash Amlani, Keshav Kumar Gupta, Huw Griffiths, Ijaz Ahmad

**Affiliations:** 1 University Hospitals Birmingham NHS Trust, Heartlands Hospital, Bordesley Green East. Birmingham, United Kingdom

## Abstract

**Background:**

Tracheostomy can be performed as an open surgical procedure, percutaneous, or hybrid and forms an important step in the management of patients infected with coronavirus disease 2019 (COVID-19) requiring weaning from mechanical ventilation. The purpose of this article is to share our experience to performing bedside surgical tracheostomy in COVID-19 patients in a safe and effective manner, whilst minimising the risk of viral transmission, to optimise patient outcomes and reduce risk to healthcare professionals.

**Material and Methods:**

As recommended by ENT UK, we prospectively established a COVID Airway Team within the ENT department at Birmingham Heartlands Hospital, consisting of four head and neck consultant surgeons to perform either open-bedside, open-theatre or percutaneous tracheostomy in COVID-19 patients. A specific stepwise method for bedside open surgical tracheostomy was based on ENT UK and British Laryngological Society recommendations.

**Results:**

Thirty patients underwent tracheostomy during the study period (14 bedside-open, 5 open-theatre, 11 percutaneous). Mean duration of mechanical intubation prior to bedside-open tracheostomy was 14.5 days. The average time for open-bedside tracheostomy was 9 minutes compared to 31 minutes for open-theatre. There were no significant tracheostomy related complications with bedside-open tracheostomy. No healthcare professional involved reported acute COVID-19 infection.

**Conclusions:**

We describe our effective, safe and swift approach to bedside open tracheostomy during the COVID-19 pandemic. Our experience demonstrated a short mean procedural time, with no tracheostomy-related complications and no reported viral transmission amongst the healthcare members involved.

** Key words:**Tracheostomy, Covid-19, SARS-CoV-2, aerosol-generating procedure.

## Introduction

The SARS-CoV-2 coronavirus disease 2019 (COVID-19) global pandemic has caused an increased number of patients requiring prolonged mechanical ventilation and subsequently requiring tracheostomy for weaning of mechanical ventilation. This has proved to be a vital part of patient care. Aerosol-generating procedures (AGP) such as intubation and tracheostomy pose significant viral transmissions risks to healthcare workers. A systematic review evaluating transmission of acute respiratory infection to health care workers during the SARS outbreak in 2003 estimated odds ratio of transmission from tracheostomy and intubation of 4.2 and 6.6 respectively (1). Several tracheostomy guidelines have subsequently emerged worldwide which have provided invaluable input including international multidisciplinary (MDT) guidance (2), and society guidance by ENT UK (3) and British Laryngology Association, amongst others.

Tracheostomy can be performed as an open surgical procedure, percutaneous, or hybrid. The decision about the optimal location for a tracheostomy procedure depends on a multitude of local factors with no available studies to suggest the superior option. The purpose of this article is to share our experience to performing bedside surgical tracheostomy in COVID-19 patients in a safe and effective manner, whilst minimising the risk of viral transmission, to optimise patient outcomes and reduce risk to healthcare professionals.

## Material and Methods

As recommended by ENT UK, we prospectively established a COVID Airway Team within the ENT department at a single centre, consisting of four ENT consultants specialising in head and neck surgery, each with over 10 years’ experience. The choice of tracheostomy method (open-bedside, open-theatre, percutaneous) and setting was agreed on a case-by-case basis via a multi-disciplinary approach with the ENT and critical care teams. This was based on patient characteristics (such as anatomical landmarks on the neck, neck extension and subsequent likelihood of success from bedside or open procedures based on surgeon and anaesthetic experience), suitability for transfer and theatre availability. Bedside surgical tracheostomy was the preferred option particularly in patients that were deemed at higher risk for instability during transfer. Our aim was to perform bedside surgical tracheostomy with maximum efficiency and safety, over the shortest time possible, to minimise the risk of viral transmission among healthcare workers while maintaining a safe outcome for the patient.

**Figure 1:**
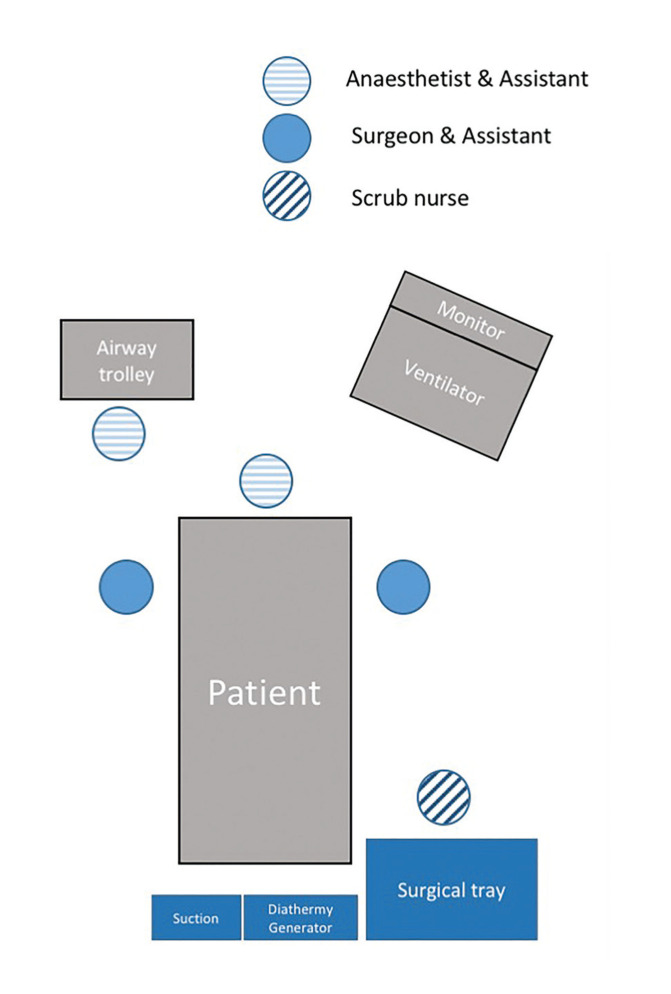
Standardised layout of patient, healthcare professionals and equipment for bedside-open tracheostomy in the intensive care unit.

Procedures were prospectively planned in semi-elective manner to ensure availability of the most experienced head and neck surgeons, senior anaesthetists and highly-skilled and familiar scrub team, whilst maintaining a minimum safe number of personnel present. All procedures were performed by no more than two ENT surgeons per procedure. Team members are all equipped with appropriate personal protective equipment (PPE) in accordance with World Health Organisation (WHO) and Public Health England (PHE) guidance. This included a PPE surgical cap, N95 mask, face shield, sterile gloves, and full-body gown. A delegated PPE officer ensured that PPE donning and doffing were correctly performed by healthcare members. All necessary equipment was prepared by experienced theatre nurse with reference to a standardised checklist and set up according to the layout demonstrated in Fig. 1.

A specific stepwise method for bedside open surgical tracheostomy was developed prospectively and based on recommendations by ENT UK and British Laryngological Society to ensure validity of our method (3). This included antiseptic prepping of the neck which was extended. The patient was pre-oxygenated with 100% FiO2. A 2-3cm median transverse incision was performed with a surgical 10 blade between 2nd and 3rd tracheal rings. Midline blunt dissection was performed with Mosquito forceps and LigaSure and retractors for access. Haemostasis was achieved with compression and bioplar cautery to achieve a bloodless field throughout. Lateral retraction and pressure with Volkmann’s retractors were used to identify strap muscles and the thyroid isthmus which was dissected bluntly (inferiorly or superiorly) to access the first three tracheal rings.

**Figure 2:**
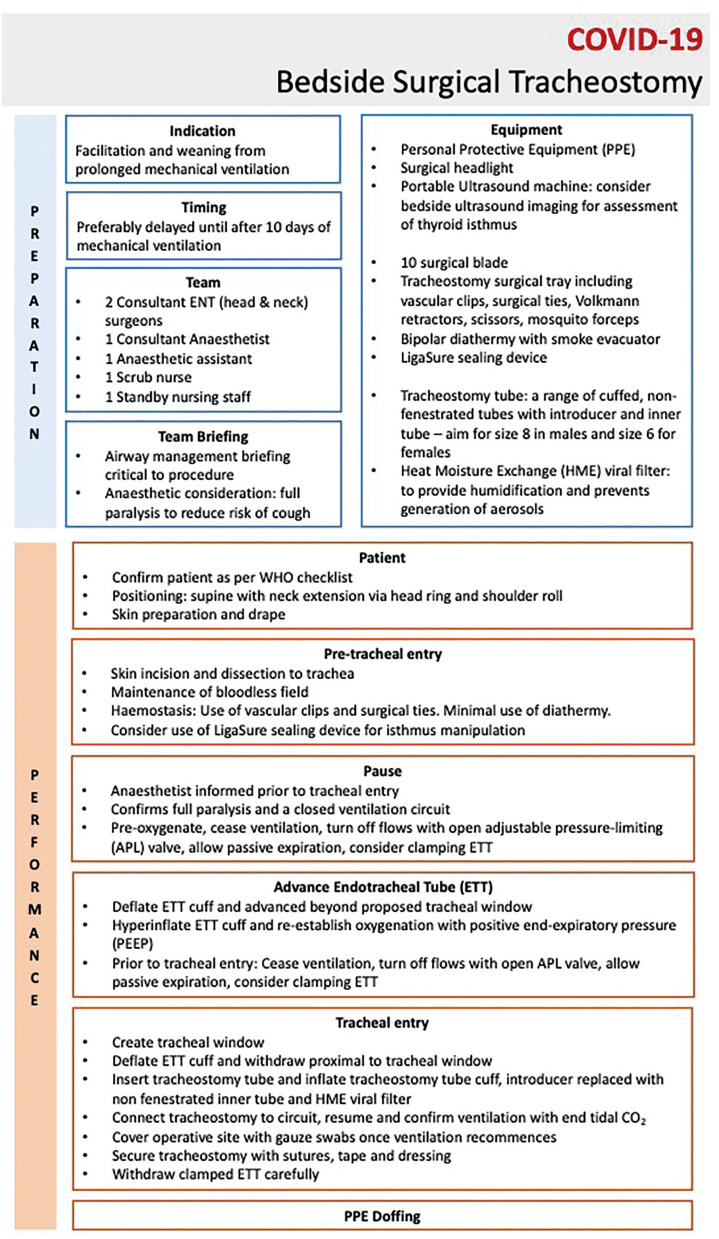
Detailed stepwise method for open-bedside surgical tracheostomy on the intensive care unit adapted from national UK guidelines (ENT-UK and the British Laryngology Association).

Following tracheal exposure, the anaesthetist slightly advanced the tracheal tube in order to place the cuff below the site of incision. Mechanical ventilation was shortly paused, the endotracheal tube cuff deflated (to minimize the risk of rupture) and the trachea was incised (rectangle) between the first and second or the second and third ring. The tracheostomy tube was lubricated and advanced into the trachea with a blunt-tip soft silicone introducer. This was then removed and replaced with an inner tube. A heat moisture exchange (HME) viral filter was attached to the tracheostomy. Mechanical ventilation was resumed after immediate reinflation of the cuff (which was tested prior to insertion) and confirmed with chest rise-and-fall, tube misting, oxygen saturations and end-tidal CO2 trace. The endotracheal tube was then removed, and the tracheostomy anchored to the skin with silk sutures. Fig. 2 provides a summary of the key steps.

Open-theatre tracheostomy and percutaneous tracheostomy were performed with a standard surgical methodology. In all cases, the operating surgeons used a Half-Face Air Purifying Respirator, visor or goggles, fluid resistant sterile theatre gown, double-gloving and head and shoe covering. All healthcare professionals were tested for COVID-19 within two weeks of each procedure. Surgeons were tested for antibodies to COVID-19 after the final included procedure. Ethical approval was not required as this is a description of a technique and outcomes deemed to be in the patient’s best interests based on national recommendations at the time.

## Results

During the COVID-19 period between 1st March 2020 and 1st June 2020, 30 patients with COVID-19 underwent a tracheostomy at our institution. This represented a total of 19 males (63.3%) and a mean age of 60.0 years (range 46 - 77 years). There were 46.7% (n = 14) undergoing a bedside open tracheostomy, 16.7% (n = 5) open tracheostomy in a theatre setting, and 36.7% (n = 11) having a percutaneous tracheostomy. The mean age of patients undergoing bedside open tracheostomy was 60.5 years old (48-69 years) where majority were male (n = 10, 71.4%) and of Asian ethnicity (n = 9, 64.3%). The mean duration of mechanical intubation prior to bedside open tracheostomy was 14.5 days. Ten patients (71.4%) who had undergone bedside open tracheostomy were still alive and had been successfully weaned from mechanical ventilation, with a mean duration of 17 days from tracheostomy to successful decannulation. In-hospital mortality was reported in 28.6% (n = 4) in the bedside open tracheostomy group, 20% (n = 5) in open-theatre group, and 18.2% (n = 11) in percutaneous group. All deaths were attributed to COVID-19 related complications and not any surgical procedure the patient had prior to their death. No significant tracheostomy related complications were reported with bedside-open tracheostomy method whilst 20% (n = 1) and 9.1% (n = 1) were reported in open-theatre and percutaneous approach respectively.

The average operating time (from skin incision to insertion of tracheostomy tube) for bedside tracheostomy in COVID-19 patients was 9 minutes. There were no intra-operative complications reported and minimal blood loss (<20ml) were achieved in all open-bedside approaches. These results are summarised in Table 1. There were no healthcare personnel involved that reported acute respiratory COVID-19 infection within two weeks of each procedure. All the head and neck surgeons had a negative outcome for COVID-19 antibodies after the final documented procedure.

**Table 1:**
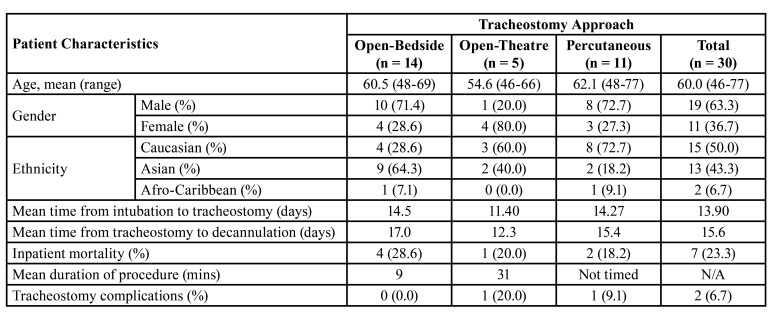
Summary of patient characteristics and tracheostomy approaches and details.

## Discussion

The results from this study demonstrate that open-bedside tracheostomy has favourable outcomes when compared to open-theatre and percutaneous approaches. The key difference was in procedure duration (mean of 9 minutes compared with 31 minutes in theatre). This was not at the expense of worse patient outcomes as there were no tracheostomy complications reported with our open-bedside approach in any patient. While the inpatient mortality was higher in the open-bedside group, no deaths were attribuTable to the surgical procedure having all been related to COVID-19. The higher in-hospital mortality and mean time to decannulation may be a reflection of the inherent selection bias in the bedside tracheostomy group as these patients are typically selected due to their comorbidities and higher risk of transfer. Further studies are needed to better understand this association. The remaining baseline patient characteristics between groups were comparable allowing these conclusions to be drawn with a fair amount of validity. It is important to note however that co-morbidities were not accounted for.

These results are in keeping with other similar studies that have demonstrated good outcomes with bedside tracheostomy (4,5). However, these studies assessed outcomes with percutaneous tracheostomy, rather than open-bedside tracheostomy which is the focus of this article. A recent study evaluated 66 bedside surgical tracheostomies and concluded that it was a safe and feasible procedure in patients with COVID-19 and to healthcare staff involved in the procedures (6). This conclusion was also reflected in a similar study conducted in Italy (7). Other studies also demonstrated positive outcomes while also showing that open methods can have less cost than percutaneous methods (8,9). In addition, a recent retrospective study showed open bedside tracheostomy in ICU had shorter operating times, similar complication rates and a crude cost saving of approximately $1900 compared to tracheostomy performed in theatre (10). Favourable outcomes were also demonstrated in another study with a lower cost compared to percutaneous dilatational tracheostomy (8).

With no established superiority of approach and location of tracheostomy procedures from the standpoint of infectious transmission, the choice is determined by balancing the risks to patients and staff and considering local expertise and resources. Performance of surgical tracheostomy in theatre requires availability of operating rooms, negative-pressure ventilation, staff and equipment, with the need for multiple disconnection and reconnection of the breathing circuit. Circuit disconnection could potentially lead to impaired oxygenation in the critically ill patient due to loss of positive end-expiratory pressure (11), and additionally increase exposure risks to additional personnel during patient transfer. A systematic review evaluating the available international guidelines for tracheostomy in COVID-19 highlighted the role of bedside tracheostomy in the Intensive Care Unit (ICU) in negative-pressure rooms (12). However, it is generally recognised that the availability of negative pressure air flow setting both in operating room and ICU is in reality limited and not a usual part of the UK hospital infrastructure. As with many institutions that were severely affected with COVID-19, multiple operating theatre rooms were converted to accommodate the saturation of ITU beds. These logistical factors, together with scarce operating room resources, favoured open surgical tracheostomy to be performed by the bedside in ICU.

The specific challenges of bedside open approach include limited space, need of transfer of surgical equipment and instruments, and suboptimal lighting and positioning. This was made more challenging due to the enhanced PPE and associated impaired communication. We find that in order to overcome these challenges, bedside open tracheostomies in the ICU should be standardised and meticulously planned with participation of highly experienced surgeons, anaesthetist and scrub team.

We followed key recommendations in minimising aerosol generation during open tracheostomy in COVID-19 patients including advancing the endotracheal tube distal to proposed site of tracheal window prior to entry, hyperinflation of endotracheal cuff, withholding ventilation at key points and covering operative site with gauze swabs when ventilation recommences (13). These factors, alongside a short mean procedural time (nine minutes) are likely to have been key contributing factors to minimising risk to healthcare workers involved in the procedure. This was reflected in no members of staff testing positive for COVID-19 and no surgeons testing antibody positive after the study completion.

A key approach in performing a safe and swift bedside tracheostomy is to ensure that major bleeding is avoided. Many of the critically ill tracheostomy candidates will be anticoagulated; making haemostasis even more crucial. Common source of bleeding is typically from the anterior jugular veins and from the encountered thyroid gland and its feeding vessels. A pre-operative ultrasound assessment can be considered in conjunction with palpation of the neck particularly in in obese patients or where anatomical landmarks are difficult to assess by palpation. It provides important anatomical information including distance from skin to trachea, identification of vulnerable structures, such as thyroid gland and blood vessels.

We acknowledge and follow the recommendations to limit the use of diathermy. The evidence surrounding risk of aerosolisation from surgical smoke plumes is still not fully understood (14), however transmission is theoretically plausible. Therefore, we opted for vascular clips and surgical ties when possible and considered diathermy on case-by-case basis; balancing the potential risk of aerosolisation with the risk of intra-operative bleeding. To mitigate theoretical viral transmission from diathermy plumes, we ensured the use of an extractor suction. Our practice also includes the use of LigaSure sealing device in cases where thyroid isthmus division is required. This approach as opposed to traditional clamping, division and ligation with transfixion sutures; is considered a less time-consuming option.

This study has strengths in the fact that it was a prospectively designed study with a strict methodology and MDT approach to each case. This allowed for consistency in terms of the peri-operative procedural planning and steps to optimise patient care. In addition, we have measured risk to staff with post-procedure testing/reporting of COVID-19 and antibody testing of surgeons. Despite this, we acknowledge the limitations of this study with no baseline co-morbidity matching between groups and small sample sizes that limit the validity of conclusions drawn. This did however represent all patients undergoing tracheostomy by any method during the first wave of the COVID pandemic (March - June 2020) at our centre. By demonstrating our methodology to open-bedside tracheostomy, we hope this can act as a pilot to reference as a successful alternative to tracheostomy in difficult circumstances with good outcomes for healthcare workers and patients.

## Conclusions

Our experience with bedside open tracheostomy in COVID-19 patients, demonstrated a short mean procedural time, with no tracheostomy-related complications and no reported viral transmission amongst the healthcare members involved. We acknowledge the technical challenges that are associated with operating outside theatre environment, however with careful planning and training, bedside approach to tracheostomy can be considered a more effective and safe approach in the COVID-19 pandemic. This study adds to the literature as one of the few reporting open-bedside tracheostomy and can act as a pilot study to build on with our transparent methodological and stepwise approach to the procedure (Fig. 2).
